# Patient preferences for HIV/AIDS therapy - a discrete choice experiment

**DOI:** 10.1186/2191-1991-3-14

**Published:** 2013-05-11

**Authors:** Axel C Mühlbacher, Matthias Stoll, Jörg Mahlich, Matthias Nübling

**Affiliations:** 1 Hochschule Neubrandenburg, Brodaer Straße 2, 17033, Neubrandenburg, Germany; 2 Medizinische Hochschule Hannover, Hannover, Germany; 3 Janssen Cilag, Neuss, Germany; 4 University of Vienna, Vienna, Austria; 5 Gesellschaft für empirische Beratung, Empirical Consulting, Denzlingen, Germany

## Abstract

**Objectives:**

An increasing emphasis on patient-centred health care and shared decision making requires an intensive consideration of patient preferences. In the present study, patient preferences regarding treatment of HIV/AIDS were explored using direct assessment and discrete choice experiment (DCE).

**Methods:**

Based on literature research about preferences of HIV/AIDS patients we conducted a qualitative pre-study. The results were used to compose a questionnaire on relevant aspects of HIV/AIDS treatment which underwent a pre-test. In the subsequent quantitative study phase presented here, the following data were collected online or on paper including socio-demographic data, SF12v2, data on HIV/AIDS, antiretroviral treatment and patient preferences for therapy characteristics using direct measurement, as well as a discrete choice experiment.

**Results:**

218 patients completed the quantitative main study, 82% of these on paper. 86% were male and the most frequent age group was between 45 and 54 years (37.6%). The SF12v2 showed a mean value of 43 points for the “mental health” component sum score. In the direct measurement the most relevant therapy characteristics were “Self-application of the drug (at home or on-the-go) possible”, “Drug has very high efficacy (reduction of viral load)” and “Long term (hidden) damage (e.g. organ damage) is unlikely”. Based on a factor analysis, six treatment characteristics were selected and used to generate eight virtual therapies. To evaluate the patient assessments a random effect logit model was employed. All of the characteristics were statistically significant predictors of the model of patient preference. The most important therapy characteristic was that the disease is not obvious for others.

**Conclusions:**

The main result is the high impact of quality of life, in particular the emotional quality of life on patient preferences on the selection of treatments. Thus, the selection of particular treatment options should be accompanied by a deliberate consideration of treatment features, which need to be considered in order to maximize patient adherence and compliance.

## Background

Progression to the acquired immune deficiency syndrome (AIDS), a disease with failure of the immune system caused by the human immune deficiency virus (HIV), characterized by certain life-threatening opportunistic infections and malignancies can be prevented by combined antiretroviral therapy (cART, [1,2]). HIV infection occurs by mucosal or parenteral body fluid contact, i.e. blood, semen, vaginal fluid, pre-ejaculate, and breast milk [3]. AIDS was first reported in 1981 [4]. At the end of 2011, an estimated 34 million people were living with HIV worldwide. The number of people dying of AIDS related causes dropped to 1.7 million in 2011, down from a peak of 2.2 million in 2005. There were 2.5 million new HIV infections in 2011. This was 20% less than in 2001 [5].

One of the factors influencing this decrease is the availability of cART, which reduces both the mortality and morbidity associated with HIV infection [3]. HIV viral suppression, reduced rates of resistance, an increase in survival, and improved quality of life have been shown to be strongly correlated with adherence to antiretroviral therapy [6,7]. Because HIV treatment is a lifelong endeavour and because many patients will initiate therapy when they are generally in good health without obvious signs or symptoms of HIV disease, adherence poses a special challenge and requires commitment from the patient and the health care team [8].

Characteristics of cART are (i) combination of three or more antiretroviral drugs, (ii) high impact of continuos administration (adherence) to avoid the development of viral resistance, (iii) high potential of drug-drug interactions between antiretrovirals or concomitant therapy, (iv) moderate to high potential for adverse events, and (v) requires durable—life long—intake. Additional psychological consequences for the patients result by social marginalisation as a HIV-infected individuum and potential stigmatisation by long term effects of cART, e.g. lipodystrophic change of body shape [8].

The present study assesses the therapy-related expectations and needs of HIV/AIDS-patients. In contrast to clinical trials concerning mainly efficacy and safety aspects, the primary goal of this study was the comprehensive evaluation of preferably all relevant aspects of treatment quality from the patients’ perspective such as effectiveness, quality of life and further treatment options. Secondary, we were interested in finding out how the study participants would judge the relevance of these criteria.

## Methods

The study population was comprised of HIV/AIDS patients, who completed paper-based and online-questionnaires. Patient recruitment was performed by the German Competence Network for HIV/AIDS (‘Kompetenznetzwerk HIV/AIDS’) located in Bochum, who helped to distribute paper-based questionnaires and the link to the online version among treatment centres. The Competence Network for HIV/AIDS is a general national research alliance, and as such includes the most important German working groups active in clinical and basic research.

### Qualitative pre-study

To ensure content validity, a qualitative pre-study was performed prior to the main study to identify important aspects of an ideal HIV/AIDS treatment. Based on a literature search (e.g. [8]), an interview guideline was composed for group discussions with HIV/AIDS patients. These patients were interviewed in four focus groups of five to nine patients.

The results of these interviews were used to design a questionnaire on relevant aspects describing the quality of HIV/AIDS treatments from the patient’s perspective. This questionnaire underwent a pre-test including 28 subjects to ensure its usability. Most of the test items appeared to be clear and comprehensible. Based on the results of the pre-test the questionnaire was finalised.

### Main survey

The main survey was performed from July 2009 until end of May 2010 using either online or paper questionnaires. Patients were contacted either in writing and were given the paper-based questionnaire version with stamped addressed envelopes, or via email/internet. Both methods were offered since older patients may not have wanted to use the online version. No personal data such as addresses, names or phone numbers were collected.

The questionnaire encompassed five main domains:

•Patient characteristics: age, gender, educational level.

•Current health status (Health related Quality of Life (QoL), SF12v2, German version of the SOEP [socio-economic panel], [9]).

•Data on HIV/AIDS and antiretroviral therapy: year of diagnosis, route of infection, year of first antiretroviral treatment.

•Direct assessment of importance of 26 items on HIV/AIDS therapy characteristics (five-point Likert scale ranging from “very important” (100 points) to “not important” (0 points))

•Assessment of current therapy using 6 therapy characteristics.

•Discrete Choice Experiment (DCE) for eliciting patient preferences using eight pairs with six characteristics each.

### Discrete choice experiment

Revealed and stated measurements are the two approaches for the measurement of patient preferences. The revealed method is based on observed decisions being evaluated, e.g. prescription data [10,11]. It shows how patients decide, but cannot explain why [12]. In stated analyses patient preferences are collected using direct questioning: patients have to assess objects with respect to several characteristics, which are presented in different combinations. Stated methods, such as conjoint and discrete choice analysis (DCE), aim to measure the influence of therapy characteristics on patient preferences [12]. The conjoint analysis is a statistical method to determine how people weigh and assess different characteristics of a product or service. The preference measurement assumes that each product or service is characterised by one or more characteristics, which in sum represent this product or service [11].

The discrete choice experiment is a choice based method, and a variant of the conjoint analysis, which was made possible through the theoretical work of Lancaster [13] and McFadden [14]. In the discrete choice experiment different therapies are presented pair-wise and the subjects have to decide for one of the options [15]. In a first step all characteristics being relevant for each target group have to be identified. These may be physician expertise, medical-technical equipment or waiting time for an appointment [16].

The relevant characteristics are then combined to define hypothetical products or treatment options. As the number of possible combinations increases exponentially, not all combinations can be presented. It is important to cover all the relevant fields when selecting the items for the DCE. Therefore, in most cases a reduced sample of alternatives is used, which, however, allows a reliable evaluation of preferences [17,18]. To appreciate the importance of possible statistical correlation between main effects and interactions, the number of combinations was reduced to a more manageable size without losing essential information through an orthogonal design (if certain assumptions about interaction effects are made). The maximum dissimilarity between therapy alternatives was achieved by generating the alternative B as exact mirror image of A (using the fold-over technique) [19].

The treatment alternatives are presented to the patient and the patient has to decide for one of the presented options. Based on the decision behaviour the relevance of the different characteristics for the decision can be calculated and described by coefficients.

### Analysis

We employed a DCE technique with eight pairwise choice situations, each with six characteristics. Respondents had to choose eight times between treatment A or B. The calculation of coefficients was performed using the maximum likelihood method. According to the underlying distribution function, different estimation methods (in most cases probit or logit estimations) were used [17,20-23]. We tested models with all the sociodemographic variables in order to explore differences between subgroups. We generated interaction-terms of each attribute with each parameter as product-terms (i.e. each attribute was interacted with age, sex and so forth). We then calculated a model with all the main effects and all the interactions of one parameter (“forced entry”). That means a single model for the whole set of variables. No strata or separate models for subgroups were applied because in that case we would have to deal with different constants. The combined “parsimonious model” was employed by testing a model with the main effects and all significant interactions of all parameters resulting in the analysis above and then reducing the model by eliminating the non significant interactions step by step. The resulting model contained all the main effects and the significant interactions. The aim of this model was to show all the subgroup effects “at a glance”.

## Results

### Patient characteristics

Between July 2009 and end of May 2010, 218 patients completed the questionnaire. Most of the patients (82%) answered via paper version and 18% online. 86% of the study participants were male, only 14% were female. The most frequent age group referred to patients between 45 and 54 years (37.6%). 22% of the patients had a lower certificate of secondary education, 33% had a general certificate of secondary school, 43% had a certificate qualifying for university admission and 2% of the patients left school without certificate.

### Disease and treatment characteristics

The patients were asked to specify the year in which their HIV/AIDS disease was diagnosed. The answers covered a range from 1982 to 2010. The mean of primary diagnosis was the year 1998 with a standard deviation of 7.3 years.

The most frequent route of infection was MSM (men having sex with men) with 55% of the patients. 12% of the patients reported heterosexual contacts, 5% iv drug use and 4% blood transfusions. For 24% of the patients the route of infection was unknown.

The question if their AIDS disease has already “broken out” was answered with “no” in 64% of the patients. The patients were asked to specify the year in which the first antiretroviral treatment was initiated for them. Answers were available in 205 of the 218 patients (94%). Eight patients specified that they have not yet received any antiretroviral treatment (5 patients with missing data). The answers on start of AIDS treatment cover a range from 1984 to 2010. The mean of the first antiretroviral treatment was the year 2001 with a standard deviation of 6.5 years.

A summary of patient data is shown in Table 1.

**Table 1:** Patient characteristics, health status and treatment characteristics

**Parameter**	**Actual data**
N	218 patients
Gender	
Male	86%
Female	14%
Completion of survey	
Paper and pencil	82%
Online	14%
Most frequent route of infection	55% MSM
Antiretrovirally treated	94%
Health status (SF12v2)	
Mental health (mean)	43 points
Physical health (mean)	50 points
Mean year of primary diagnosis (SD)	1998 (7.3)
Mean year of first AIDS treatment (SD)	2001 (6.5)

### Direct assessment of AIDS therapy characteristics

The respondents had to rate the importance of 26 therapy characteristics. The mean assessments of importance are included in Table 2. All except 3 of the items concerning quality of treatment were of relatively high mean importance (>70 points) for the patients. These ceiling effects are not surprising, since only aspects were presented that were rated as being important according to the literature and the qualitative pre-study.

**Table 2:** Mean importance of therapy characteristics and factor weightings for the 6-factor model (items sorted by weighting)

**Item No.**	**Therapy characteristic (short form)**	**Mean importance**	**1**	**2**	**3**	**4**	**5**	**6**
10	Self-application of the drug is possible	94		.569		.312		.328
1	Drug has very high efficacy	94				.716		
18	Long term (hidden) side effects unlikely	92						.620
2	Maximum prolongation of life expectancy	90				.688		
4	Drug does not generate resistance	90			.383	.599		
20	Drug does not affect appearance	89		.317			.410	
19	Drug improves physical state	89	.400					.513
3	Drug allows further therapy options	88				.716		
16	Rarely occurring nausea	86					.868	
17	Rarely occurring diarrhoea	86					.842	
14	Drug can be taken along without problems	85		.659				
7	Long duration of efficacy	85		.384	.315	.381		
6	Long term use of the drug is possible	84			.323	.419		
23	Drug allows improved mobility	82	.789					
12	Flexible application	82		.729				
21	Emotional and mental state improved	81	.749					
5	Can be used also in case of comorbidities	80			.488	.386		
13	Simple application: only few tablets	79		.736				
22	Social contact opportunities improved	78	.820					
24	Drug does not cause additional costs	75	.492		.539			
11	Once daily application	73		.703	.362			
15	Inconspiciuous drug intake	73	.318	.404				−.462
25	Treatment does not require much time	71	.600	.345				
9	Dosing of drug may vary	60			.742			
8	Therapy-free intervals possible	51			.713			
26	Pregnancy allowed	30			.524			

### Factor analysis

Using factor analysis higher-level dimensions were found to summarise the importance assessments. Using a Principal Components Analysis (PCA) including varimax rotation, six factors were found declaring 60.2% of the variance of the 26 items. The rotated factor matrix is shown in Table 2.

Five of the six factors may be characterised by the following semantic descriptions: factor 1 covers the social situation, everyday life and social quality of life, factor 2 flexibility of medication, factor 4 efficacy and life expectancy, factor 5 physical and emotional quality of life and factor 6 long term side effects. Factor 3 covers several aspects combining dosing, treatment interruptions, extra payment and pregnancy.

The most relevant therapy characteristics represented by mean assessments of more than 90 points were:

Self-application of the drug is possible,

Drug has very high efficacy,

Long term (hidden) side effects are unlikely.

### Assessment of current therapy

The first step for the patients was to assess their current therapy using these six therapy characteristics. In most cases the patients assessed their current therapy as positive demonstrated by assessments “yes”, “rather yes” or “probably yes” in 64% to 96% of the patients. In detail, more than 90% of the patients assessed their therapy to provide maximal increase of life expectancy and allow participation in social life and 84% of the patients assessed that their therapy avoids that the disease is obvious for others.

### Socio-demographic baseline characteristics

Using analyses of variance, the importance assessments were evaluated for effects of socio-demographic baseline characteristics of the patients: older patients prefer drugs which can be dosed according to current health status. For female patients it is important that pregnancy is possible. If HIV/AIDS has already broken out, treatment aspects such as therapy free intervals, flexible dosing, less tablets and discrete intake are more important than for average patients.

### Preferences in the discrete choice experiment

Discrete choice experiments are limited to the use of only a few characteristics. Based on the pre-study and on the results of the factor, six characteristics were selected and described by a positive and a negative pole helping to distinguish clearly between treatments (see Table 3). As these six characteristics are corresponding to the 6-factor-solution of the factor analysis, they were assumed to cover the relevant range of treatment characteristics. The final definition of the items “long term side effects” (<20% vs. ≥20% of patients) and “flexibility of dosing” (max. 3 vs. ≥4 tablets/day) was due to clinical expertise.

**Table 3:** Treatment characteristics for discrete choice experiment

**Characteristics**	**Positive pole (+)**	**Negative pole (−)**
Life expectancy	maximal increase	moderate increase
Long term side effects	improbable (<20% of patients)	possible (≥20% of patients)
Flexibility of dosing	max. 3 tablets/day	≥4 tablets/day
Physical quality of life	diarrhoea or nausea less frequent	diarrhoea or nausea more frequent
Emotional quality of life	disease not obvious for others	disease obvious for others
Social quality of life	participation in social life possible	participation in social life restricted

### Generation of pairs

Based on the six therapy characteristics eight virtual therapies were generated. These eight therapies were presented to the patients in eight pairs from which the patient had to select one of the two therapies (A or B).

In total, 1604 valid observations were available. Some of the respondents did not evaluate any or all pairs. On average, out of 214 respondents who finished at least one pair comparison, 7.5 pairs were available (range 1–8).

In both of the chosen treatments physical, emotional and social quality of life were positive, combined either with maximum increased life expectancy (pair 8) or less long term damage (pair 4). A random effect logit model was created, which takes the partial dependency of observations from the same person concerning the parameter estimation into account. Estimated coefficients and their standard errors are shown in Table 4.

**Table 4:** Results of random effects logit model (discrete choice experiment; negative pole as reference group)

**Characteristics**	**Coefficient**	**SE (coeff.)**	**Sig.**	**Partial log likelihood**
Life expectancy: maximal increase	0.735	0.152	***	−717.7
Long term side effects improbable (<20%)	0.408	0.147	**	−709.5
Flexibility of dosing: max. 3 tablets/day	0.454	0.151	**	−710.1
Physical quality of life: diarrhoea, nausea less frequent	1.611	0.152	***	−769.4
Emotional quality of life: disease not obvious for others	2.984	0.153	***	−981.7
Social quality of life: participation in social life possible	1.140	0.153	***	−735.4
Model constant	−3.726	0.214	***	

***: p < 0.001, **: p < 0.01.

Model parameters:

Wald Chi2 (df = 6) = 470.81.

Log likelihood = −705.7.

Prob > Chi2 = 0.0000 (i.e. p < 0.001, ***).

Prob ≥ Chi2 = 1.000.

All of the treatment characteristics were statistically significant predictors of the model of patient preferences. The characteristic “Emotional quality of life” was the most important, followed by “Physical quality of life” and “Social quality of life”. Less important but still statistically significant were the items “Life expectancy”, “Flexibility of dosing” and “Long term side effects improbable”.

A supplementary partial log likelihood analysis as proposed by Lancsar et al. [24] yielded a similar hierarchy as the interpretation based on the item coefficients.

In additional evaluations, socio-demographic parameters were analysed. Subgroup effects were found particularly for “Emotional quality of life”, which was significantly less important for older patients, patients with longer duration of infection and patients being under therapy for a longer period. MSM patients, however, found this characteristic more important, as well as the characteristic “Physical quality of life”. For patients with a higher level of education maximum increase of life expectancy was more important.

Each pair comparison was accompanied by an assessment of whether the patient would change his or her current therapy in favour of the treatment of choice of the corresponding pair. “No change” answers ranging between 73% and 89% demonstrate a high degree of satisfaction with the current therapy.

At the end of the questionnaire, the patients were asked to give an assessment of the degree of difficulty they had in performing the paired comparisons in the DCE. 8% found it “very difficult”, 29% said it was “rather difficult”, 36% gave a medium ranking (“some comparisons more difficult than others”), 18% thought it was “not very difficult” and 8% claimed they had “no problems at all” in completing the DCE. This means that overall, the DCE can be considered as feasible from the patients’ point of view.

## Discussion

The patient view and desires in healthcare decisions (e.g. treatment characteristics) are often not sufficiently considered. However, in times of limited healthcare resources, both shared decision making and patient involvement in treatment decisions have been encouraged in recent years. This requires an understanding of patient priorities concerning treatment decision making.

The present study collects and depicts the preferences of HIV/AIDS patients in Germany regarding their therapy. Based on a qualitative study to ensure content validity, a quantitative preference measurement was performed including a direct assessment and a choice-based measurement of patient preferences (DCE). This latter technique allows the estimation of the relative importance of different aspects of care and the trade-offs between these aspects, while the direct assessment allows the inclusion of more aspects. Hence, both methods should be used in a combined way.

The current patient sample includes a proportion of 43% patients with certificate qualifying for university admission. Compared to the KompNet data set of more than 9,000 patients with HIV/AIDS [25] presenting a rate of 33% of the male patients with higher education entrance qualification [26], the current patient sample seems to have an increased rate of patients with higher level of education. However, as the educational level seems to have only limited effect on the preference structure, a possible bias may be of minor effect. In total, there is no reason to assume that the documented patient preferences will be biased substantially by sample effects.

The current health status was assessed by the SF12v2 questionnaire (version 2 for the socio-economic panel, SOEP). The mean value of 43 points for the “mental health” component summary score is 0.7 standard deviations below the German average. This indicates that the adults with HIV/AIDS of this sample assess their mental health as worse compared to the assessments of the German population norm. In contrast, the mean value for the “physical health” component summary score of 50 points indicates an assessment of the HIV/AIDS patients being not different from the population norm.

The results of the SF12v2 subscales confirm this picture: in particular the subscales “role emotional” and “social functioning” which both belong to the “mental health” component summary score fall quite clearly below the average of the population norm.

In the main study patients had to assess a total of 26 treatment characteristics which had been shown to be relevant by literature research and the qualitative pre-study. As expected, the results demonstrate a ceiling effect of the assessments, which were found predominantly in the upper range of the scales, perhaps because mainly important characteristics were presented as a result of the qualitative pre-study. The top of the priority list is marked by needs such as high efficacy, the avoidance of long term and short term side effects, but also by the improvement of emotional and social status.

In a second step the preferences were evaluated using a discrete choice experiment. During this experiment the patients had to choose 8 times between two fictitious treatments, each characterized by 6 characteristics. The used analysis model allowed determining the amount to which each characteristic contributed to the treatment choice.

The highest relevance for the treatment choice of the patients was found for emotional quality of life being mainly characterized by the characteristic that the disease was not obvious for other persons. The next relevant patient-relevant outcome from the patient point of view were the avoidance of physical impairments such as diarrhoea and vomiting and the facilitation to participate in social life. Being still statistically significant, the characteristic “maximum increase of life expectancy” followed at some distance. The reduction of the risk of long term side effects (<20% vs. >20%) as well as the flexibility of dosing (intake of 3 tablets per day vs. 4 or more tablets) seemed to be less important for the treatment choice.

The results of the DCE correspond partly to the direct assessment where emotional, physical and social quality of life were also in the upper range of needs. Surprisingly, however, the characteristics “maximum increase of life expectancy” and “avoidance of the risk of long term damage” seem to be far less important in DCE than in the direct assessment of importance. DCE results emphasise to see HIV/AIDS more as a chronic disease than as directly life threatening. This corresponds well to impairments of mental health found in the SF-12 questionnaire. As a consequence, using quality of life arguments rather than efficacy and safety aspects may increase therapy adherence of the patients. However, these arguments differ from the known aspects like pill count, dosing frequency and adverse events shown by Stone et al. [27] or resistance, regimen convenience and sleep disturbance shown by Beusterien et al. [28]

The subgroup analyses have shown that the most important characteristic “emotional quality of life” was weighted even higher in some subgroups such as younger patients, MSM patients (together with physical quality of life) and patients with shorter duration of infection and treatment. Presumably, in these patients the understanding of HIV/AIDS as a long term disease is intensified, highlighting aspects of a life being impaired by the disease as little as possible. Furthermore, patients with a higher level of education (“Abitur”, i.e. high-school diploma) assessed the characteristic “maximum increase of life expectancy” as relatively more important.

Based on these considerations, some limitations of the current study may be discussed. Because the DCE is only manageable using a few characteristics and pairs to be compared, decisions need to be made during construction of comparisons. In principle, the composition of characteristics reflects the factor structure found in the direct measurement. In doing this, however, some problems remained: for example, dosing aspects were presented as maximum 3 tablets per day versus more, whereas the direct assessment revealed more facets of application such as dosing according to current health status, treatment-free periods or self-application. As these and some more aspects of application affected at least two factors in the factor analysis, the characteristic “dosing flexibility” may have been defined too simply to reflect the complexity of dosing characteristics. Furthermore, the definition of poles of the characteristics may have influenced the preference decisions: the item “increase of life expectancy” was presented as “increase” versus “maximum increase”. Maybe the perceived difference between these two poles was too small to prefer this characteristic against others. Thus, the somewhat surprising low importance of this efficacy parameter may be the consequence of too close definitions of the poles of this characteristic.

Although our recruitment procedure did not guarantee representativeness, the sample fits quite well to the expected picture of HIV/AIDS patients. For instance, the rate of female patients (14%) corresponds well to the rate of the KompNet cohort (15%) [26], and the proportion of patients with MSM (men having sex with men) as source of infection of 55% in the current sample corresponds well to the rates of 56% for the ClinSurv HIV cohort and 55% of national German HIV surveillance data reported by Bätzing-Feigenbaum et al. [29]. The proportion of 94% of patients reporting being treated with antiretroviral therapy at some time during their HIV/AIDS disease in the current sample appears to be higher than in the total sample of HIV/AIDS patients, being specified between 75% and 80% by the RKI Bulletin [30]. However, for our research question the quality of answers may even benefit from a higher rate of treated patients.

## Conclusion

In summary, direct assessments as well as DCE contribute important findings to the knowledge of preference structures of patients suffering from HIV/AIDS. The main result of the current study may be the fact that it is very important for HIV/AIDS patients that their antiretroviral therapy supports their quality of life, in particular the emotional quality of life. This might reflect a paradigm shift in the grading of preferences since more convenient and less toxic options for cART became available within the last decade. Thus, the selection of a particular treatment regimen should be accompanied by a deliberate consideration of features of possible treatment options, which need to be considered in order to maximise patient adherence and compliance to the selected treatment.

## Consent

Written informed consent was obtained from the patient for publication of this report and any accompanying images. The participant had the right and chance to end the study at any time. The decision not to participate or to withdraw from the study did not involve any penalty.

## Competing interests

The research project was financially supported by Janssen-Cilag. JM is employed by Janssen-Cilag. AM, MN and MS declare that they have no competing interests.

## Authors’ contributions

AM and MN designed and carried out the empirical study. MS acted as a clinical adviser and participated in the coordination of the study. JM contributed to the draft of the paper. All authors read and approved the final manuscript.

## References

[B1] Pichenot M Deuffic-Burban S Cuzin L Efficacy of new antiretroviral drugs in treatment-experienced HIV-infected patients: a systematic review and meta-analysis of recent randomized controlled trials HIV Med 2012 3 148 155 22107456 10.1111/j.1468-1293.2011.00953.x

[B2] Thompson MA Aberg JA Hoy JF Antiretroviral treatment of adult HIV infection: 2012 recommendations of the International Antiviral Society-USA panel JAMA 2012 3 387 402 10.1001/jama.2012.7961 22820792

[B3] Case KK Ghys PD Gouws E Understanding the modes of transmission model of new HIV infection and its use in prevention planning Bull World Health Organ 2012 3 831 838A 10.2471/BLT.12.102574 23226895 PMC3506404

[B4] Centers for Disease Control and Prevention (CDC) Pneumocystis pneumonia – Los Angeles MMWR Morb Mortal Wkly Rep 1981 3 250 252 6265753

[B5] Global Report UNAIDS Report on the global AIDS epidemic (PDF) http://www.unaids.org/en/media/unaids/contentassets/documents/epidemiology/2012/gr2012/20121120_UNAIDS_Global_Report_2012_en.pdf, downloaded on January, 27^th^ 2013

[B6] World Health Organization (WHO) Adherence to long term therapies – evidence for actionhttp://www.who.int/chp/knowledge/publications/adherence_full_report.pdf, 2003, downloaded on May 3rd 2012

[B7] Grierson J Koelmeyer RL Smith A Adherence to antiretroviral therapy: factors independently associated with reported difficulty taking antiretroviral therapy in a national sample of HIV-positive Australians HIV Med 2011 3 562 569 10.1111/j.1468-1293.2011.00928.x 21554524

[B8] DHHS panel Guidelines for the use of antiretroviral agents in HIV-1-infected adults and adolescents (PDF) 2012 Department of Health and Human Services Panel on Clinical Practices for Treatment of HIV Infection 1 240 http://aidsinfo.nih.gov/contentfiles/lvguidelines/adultandadolescentgl.pdf, downloaded on January 27^th^ 2013

[B9] Nübling M Andersen H Mühlbacher AC Entwicklung eines Verfahrens zur Berechnung der körperlichen und psychischen Summenskalen auf Basis der SOEP - Version des SF 12 (Algorithmus). Data Documentation 16 2006 Berlin: Deutsches Institut für Wirtschaftsforschung (DIW) http://www.diw.de/documents/publikationen/73/diw_01.c.56544.de/diw_sp0006.pdf, downloaded on May 3rd 2012

[B10] Train K Discrete choice methods with simulation 2003 Cambridge: Univ. Press

[B11] Heidbrink M Reliabilität und Validität von Verfahren der Präferenzmessung. Ein meta-analytischer Vergleich verschiedener Verfahren der Conjoint-Analyse 2006 Universität Münster, Dissertation https://miami.uni-muenster.de/servlets/DerivateServlet/Derivate-3827/diss_heidbrink.pdf, downloaded on May 3rd 2012

[B12] Bridges J Onukwugha E Johnson F Patient preference methods–a patient centered evaluation paradigm ISPOR Connections 2007 3 4 7

[B13] Lancaster KJ A new approach to consumer theory Journal of Political Economy 1966 3 132 157 10.1086/259131

[B14] McFadden D Zarembka P Conditional logit analysis of qualitative choice behavior Frontiers in Economics 1974 New York: Academic Press

[B15] Ben-Akiva ME Lerman SR Discrete choice analysis: theory and application to travel demand 1985 Cambridge: MIT Press

[B16] Mühlbacher AC Juhnke C Bethge S Experts’ judgment on patient-centered coordinated care Value Health 2010 3 7 A337

[B17] Telser H Nutzenmessung im Gesundheitswesen: Die Methode der Discrete-Choice-Experimente 2002 Hamburg: Kovac

[B18] Street DJ Burgess L The construction of optimal stated choice experiments, theory and methods 2007 New Jersey: Wiley

[B19] Bradley M User’s Manual for SPEED Version 2.1 Stated preference experiment editor and designer 1991 Hague Consulting Group: Hague

[B20] Louviere JJ Hensher DA Swait JD Stated choice methods: analysis and application 2000 Cambridge: Univ. Press

[B21] Hensher DA Rose JM Greene WH Applied choice analysis: a primer 2005 Cambridge: Univ. Press

[B22] Bridges JF Kinter ET Kidane L Things are looking up since we started listening to patients: trends in the application of conjoint analysis in health 1982-2007 Patient 2008 3 273 282 10.2165/1312067-200801040-00009 22272995

[B23] Ryan M Gerard K Amaya-Amaya M Using discrete choice experiments to value health and health care 2008 Dordrecht: Springer

[B24] Lancsar E Louviere J Flynn T Several methods to investigate relative attribute impact in stated preference experiments Soc Sci Med 2007 3 1738 1753 10.1016/j.socscimed.2006.12.007 17257725

[B25] Jansen K Brockmeyer NH Hahn M Epidemiological composition, clinical and treatment characteristics of the patient cohort of the German competence network for HIV/AIDS Eur J Med Res 2009 3 415 425 19748848 10.1186/2047-783X-14-10-415 PMC3352224

[B26] KompNet HIV/AIDS Kompetenznetz HIV/AIDS. Aktuelle Daten 2012 http://www.kompetenznetz-hiv.de/projekte/1180.htm, downloaded on May 3rd 2012

[B27] Stone VE Jordan J Tolson J Perspectives on adherence and simplicity for HIV-infected patients on antiretroviral therapy. Self-report of the relative importance of multiple attributes of highly active antiretroviral therapy (HAART) regimens in predicting adherence J Acquir Immune Defic Syndr 2004 3 808 816 10.1097/00126334-200407010-00007 15213564

[B28] Beusterien KM Dziekan K Flood E Understanding patient preferences for HIV medications using adaptive conjoint analysis: feasibility assessment Value Health 2005 3 453 461 10.1111/j.1524-4733.2005.00036.x 16091022

[B29] Bätzing-Feigenbaum J Kollan C Kühne A Cohort profile: the German ClinSurv HIV project – a multicentre open clinical cohort study supplementing national HIV surveillance HIV Med 2011 3 269 278 10.1111/j.1468-1293.2010.00879.x 20955355

[B30] Robert-Koch-Institut (RKI) HIV-Infektionen und AIDS-Erkrankungen in Deutschland Epidemiologisches Bulletin 2011 3 172 198

